# Supplementary material for: Oncological Outcomes and Patterns of Recurrence after the Surgical Resection of an Invasive Intraductal Papillary Mucinous Neoplasm versus Primary Pancreatic Ductal Adenocarcinoma: An Analysis from the German Cancer Registry Group of the Society of German Tumor Centers
Source: Cancers (Basel). 2024 May 26;16(11):2016. doi: 10.3390/cancers16112016 (PMC11171342; doi:10.3390/cancers16112016)
Supplement: Supplementary file 1 [file cancers-16-02016-s001.zip › cancers-3013327-supplementary.pdf]

Supplementary Information

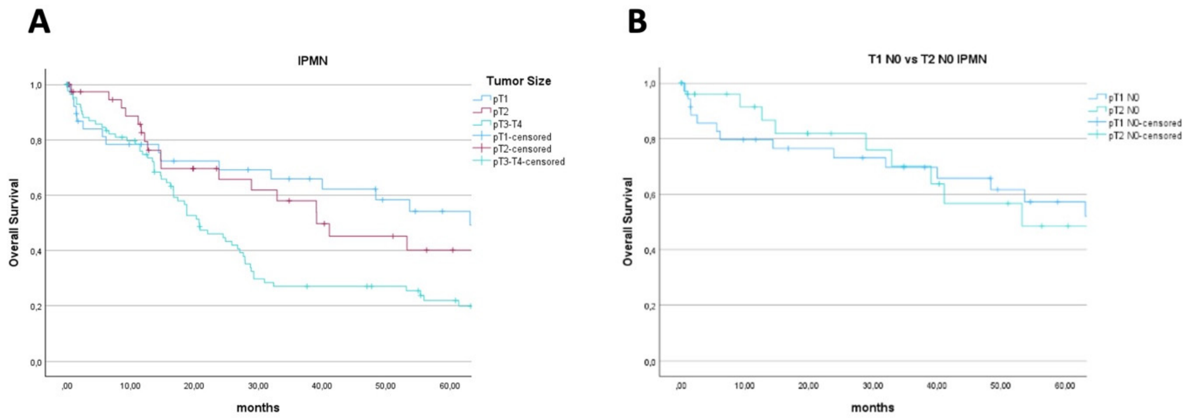

Figure S1: Survival analyses in T1 and T2 invasive IPMN.

- **(A) Kaplan Meier Plot for overall survival according to T-stage in IPMN.**

  patients with T1 and T2 invasive IPMNs exhibited comparable median overall survival (OS) durations (39 and 34 months, p=0.995) compared to a median OS of 20 months in T3-4 tumors (T1 vs T3-4 p=0.018, T2 vs T3-T4 p=0.028, respectively)

- **(B) Kaplan Meier Plot for overall survival in T1N0 and T2N0 in IPMN.**

  Patients with T1 N0 and T2 N0 invasive IPMN demonstrated similar OS rates (p=0.807).

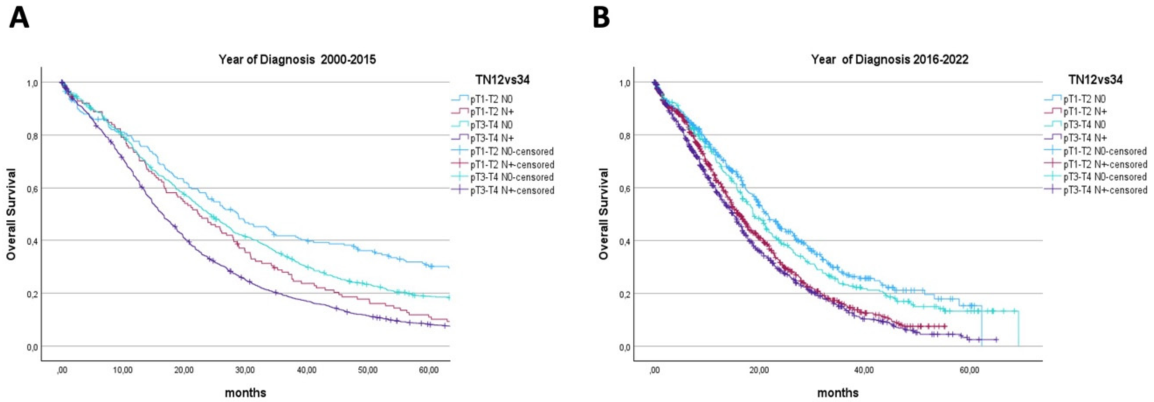

Figure S2: Sensitivity Analysis: Survival analyses before and after UICC classification changes.

Kaplan Meier Plot for overall survival before 2016 (A) and after 2016 (B) by merging tumor size and lymph node metastasis into T1-T2 N0 , T1-T2 N+, T3-T4 N0 and T3-T4 N+. $p<0.001$ respectively.

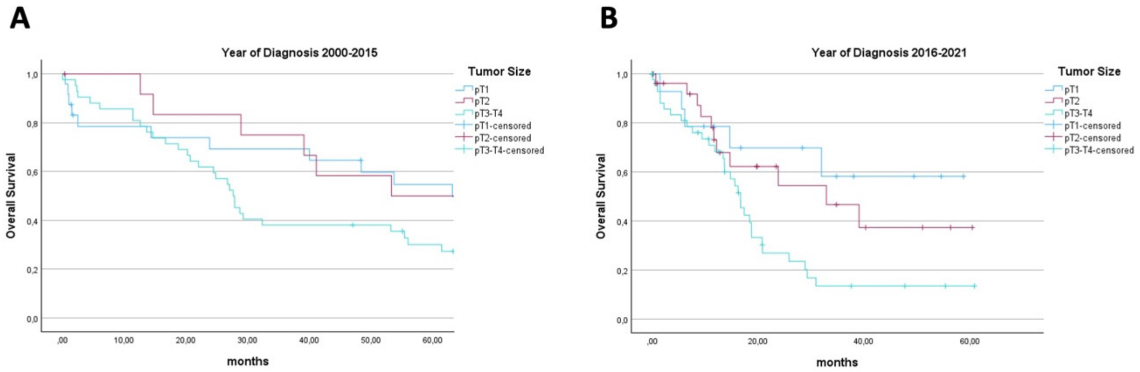

Figure S3: Sensitivity Analysis: Survival analyses before and after UICC classification changes.

Kaplan Meier Plot for overall survival according to T-Stage in IPMN before 2016 (A) and after 2016 (B) (p=0.047 and p<0.001, respectively).

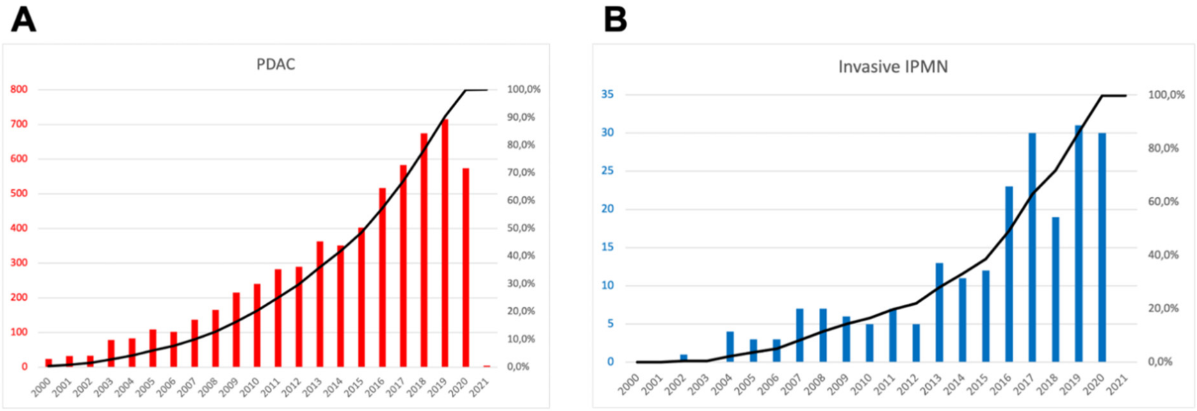

Figure S4: Diagnoses over the investigation period.

Yearly diagnoses of PDAC (A) and invasive IPMN (B) with absolute numbers of diagnoses per year (bars) and accumulation until reaching the study population in percent (black line).

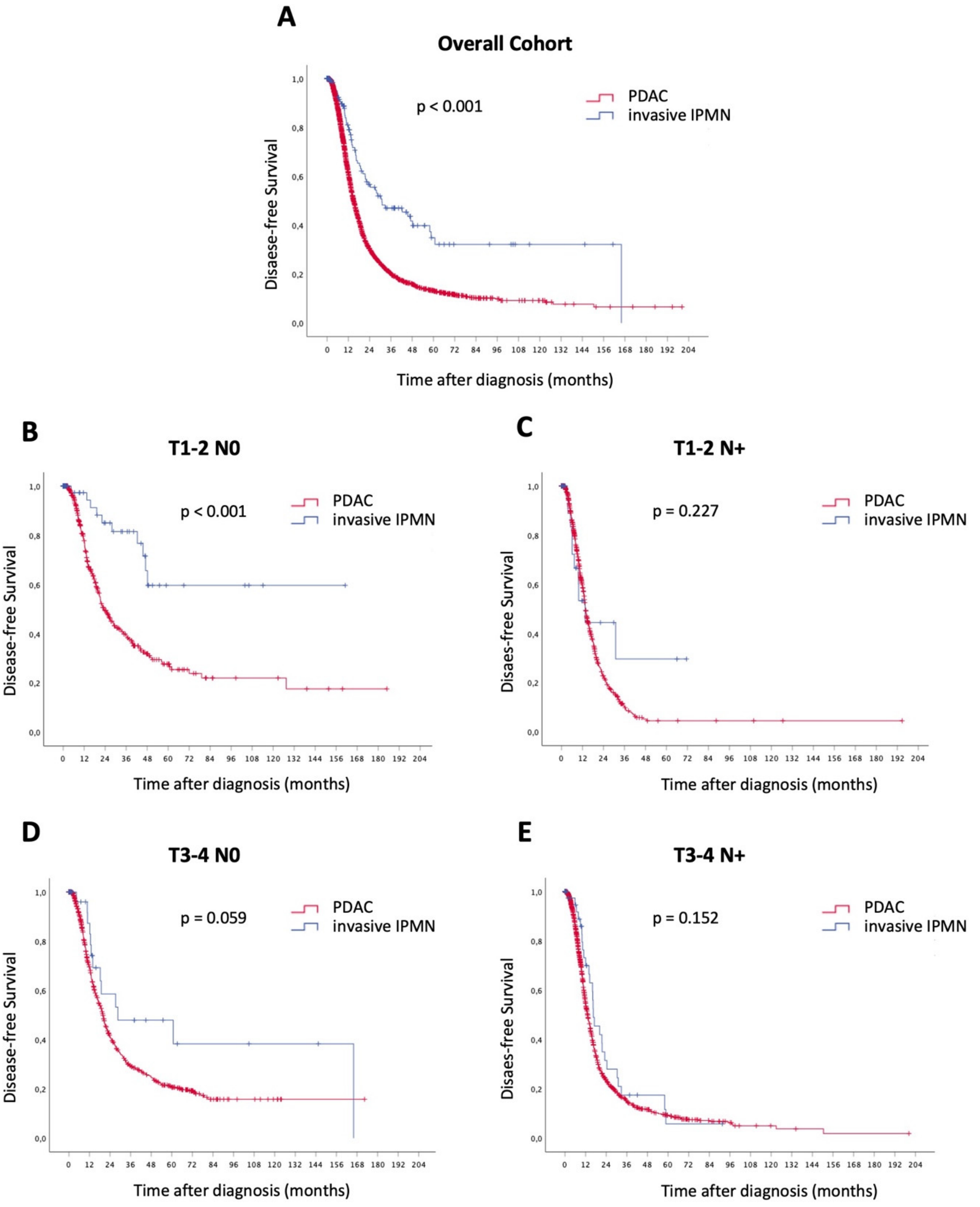

Figure S5: Disease-free Survival in resected PDAC and invasive IPMN

Disease-free Survival of resected PDAC (red line) vs. invasive IPMN (blue line), in the overall cohort (A), and according to the tumor size and nodal stage (B–E).

Supplementary Table S1: Overall- and Disease-free Survival fractions after surgical resection in PDAC patients compared to invasive IPMN.

	PDAC	Invasive IPMN	p-value
**Overall Survival**			
1-year	66%	78%	< 0.001
3-years	22%	42%	
5-years	10%	33%	
Median (months)	19 ± 1	29 ± 6	
**Disease-free survival**			
1-year	69%	85%	< 0.001
3-years	23%	50%	
5-years	15%	38%	
Median (months)	15 ± 1	31 ± 18	

Supplementary Table S2: Locations of metachronous metastases following resection of PDAC and invasive IPMN.

	PDAC	Invasive IPMN
	n = 1950	*n = 50*
**Liver**	781 (41.1 %)	21 (42.0 %)
**Lungs**	270 (13.8 %)	13 (26.0 %)
**Peritoneum**	354 (18.2 %)	6 (12.0 %)
**Pleura**	23 (1.2 %)	1 (2.0 %)
**Adrenal**	1 (0.1 %)	0
**Bones**	40 (2.1 %)	0
**Skin**	8 (0.4 %)	0
**Brain**	4 (0.2 %)	0
**Other single sites**	36 (1.8 %)	2 (4.0 %)
**Multiple location**	433 (22.2 %)	7 (14.0 %)
